# Association of lower body mass index with increased glycemic variability in patients with newly diagnosed type 2 diabetes: a cross-sectional study in China

**DOI:** 10.18632/oncotarget.17111

**Published:** 2017-04-14

**Authors:** Jian Wang, Rengna Yan, Juan Wen, Xiaocen Kong, Huiqin Li, Peihua Zhou, Honghong Zhu, Xiaofei Su, Jianhua Ma

**Affiliations:** ^1^ Department of Endocrinology, Nanjing First Hospital, Nanjing Medical University, Nanjing, China; ^2^ Nanjing Maternity and Child Health Care Institute, Nanjing Maternity and Child Health Care Hospital, Obstetrics and Gynecology Hospital Affiliated to Nanjing Medical University, Nanjing, China

**Keywords:** body mass index, glycemic variability, continuous glucose monitoring, obesity, postprandial glucose excursion

## Abstract

Previous studies have indicated that the pathogenesis of diabetes differs between obese and lean patients. We investigated whether newly diagnosed Chinese diabetic patients with different body mass indices (BMIs) have different glycemic variability, and we assessed the relationship between BMI and glycemic variability. This was a cross-sectional study that included 169 newly diagnosed and drug-naïve type 2 diabetic patients (mean age, 51.33 ± 9.83 years; 110 men). The clinical factors and results of the 75-g oral glucose tolerance test were all recorded. Glycemic variability was assessed using continuous glucose monitoring. Compared with overweight or obese patients (BMI ≥ 24 kg/m2), underweight or normal-weight patients (BMI < 24 kg/m2) had higher levels of blood glucose fluctuation parameters, particularly in terms of mean amplitude of glycemic excursion (MAGE 6.64 ± 2.38 *vs*. 5.67 ± 2.05; *P* = 0.007) and postprandial glucose excursions (PPGEs) (PPGE at breakfast, 7.72 ± 2.79 *vs*. 6.79 ± 2.40, *P* = 0.028; PPGE at lunch, 5.53 ± 2.70 *vs*. 5.07 ± 2.40, *P* = 0.285; PPGE at dinner, 5.96 ± 2.24 *vs*. 4.87 ± 2.50, *P* = 0.008). BMI was negatively correlated with glycemic variability (*r* = −0.243, *P* = 0.002). On multiple linear regression analyses, BMI (*β* = −0.231, *P* = 0.013) and Insulin Secretion Sensitivity Index-2 (*β* = −0.204, *P* = 0.048) were two independent predictors of glycemic variability. In conclusion, lower BMI was associated with increased glycemic variability, characterized by elevated PPGEs, in newly diagnosed Chinese type 2 diabetic patients.

## INTRODUCTION

With the rapid economic growth, increase in life expectancy, and changes in lifestyle, the prevalence of diabetes in China has increased significantly in recent decades. In 1980, the prevalence of diabetes in the Chinese population was less than 1% [1]. In 2010, the national survey [2] estimated the prevalence of diabetes to be 11.6%, which represented up to 113.9 million Chinese adults with diabetes. Diabetes is a major risk factor for cardiovascular disease, which is the leading cause of death in China [3]. Therefore, diabetes has become a major public health problem in the country.

Insulin resistance and impaired insulin secretion are the two main components in the pathophysiology of type 2 diabetes mellitus (T2DM); the contributions of these factors are thought to differ between Chinese and Caucasians [4]. In Caucasians, obesity and its close association with insulin resistance is a major predictor of T2DM, whereas half of T2DM patients in China have normal body weight [5]. Chan et al. [6] reported that the predominant mechanism in lean Chinese diabetic patients was impaired insulin secretion, whereas that for obese subjects was insulin resistance. Considering the different pathogenesis of T2DM between obese and lean patients, assessment of the glycemic characteristics in diabetic patients with different body mass index (BMI) is important.

Glycated hemoglobin (HbA1c), which reflected the blood glucose levels over the 2-3 months, has been considered as the gold standard for blood glucose control, but it does not represent the individual's current glycemic status. Alternatively, continuous glucose monitoring (CGM) provides information about the direction, magnitude, duration, frequency, and causes of glucose fluctuation to help physicians and patients detect nocturnal hypoglycemia, dawn phenomenon, and postprandial hyperglycemia [7]. Multiple studies have shown that glucose variability is a potential risk factor for diabetes complications [8–10]. Therefore, it is necessary to evaluate the features of glucose variations in diabetic patients, in addition to the assessment of HbA1c; however, it remains unknown whether newly diagnosed Chinese diabetic patients with different BMI have different features of glucose variations.

The aim of this study was to determine the differences in the characteristics of glucose fluctuation between underweight or normal-weight patients and overweight or obese patients, as well as the relationship between BMI and glycemic variability in newly diagnosed Chinese T2DM patients.

## RESULTS

### Clinical and biochemical characteristics

The 169 newly diagnosed T2DM patients had a mean age of 51.33 ± 9.83 years and were grouped into underweight or normal-weight patients (*n* = 57) and overweight or obese patients (*n* = 112). As shown in Table 1, compared with underweight or normal-weight patients, overweight or obese patients were more likely to have higher waist circumference (WC), alanine aminotransferase (ALT), low-density lipoprotein cholesterol (LDL-C), uric acid (UA) and proportion of patients with hypertension. All other characteristics were not significantly different between the two groups.

**Table 1:** Clinical and biochemical characteristics of the study patients

Characteristics	Total (*n*=169)	Underweight or Normal-weight (BMI<24, *n*=57)	Overweight or Obesity (BMI≥24, *n*=112)	*P*-Value
Age (years)	51.33±9.83	51.86±9.53	51.06±10.02	0.620
Male, n (%)	110(65.01%)	33(57.89%)	77(68.75%)	0.162
Current smokers (n (%))	61(36.09%)	16(28.07%)	45(40.18%)	0.121
Current alcohol (n (%))	32(18.93%)	9(15.80%)	23(20.54%)	0.457
Hypertension (n (%))	68(40.24%)	15(26.32%)	53(47.32%)	0.008
Family history of T2DM (n (%))	63(37.28%)	18(31.58%)	45(40.18%)	0.274
BMI (kg/m2)	25.36±3.09	22.28±1.46	26.93±2.46	<0.001
WC (cm)	87.54±7.56	81.96±5.27	90.38±6.96	<0.001
ALT (u/L)*	25.00 (16.00, 40.50)	19.00 (13.00, 33.00)	26.50 (19.25, 44.75)	0.003
AST (u/L)*	20.00 (15.00, 28.00)	18.00 (14.00, 25.00)	20.00 (15.00, 30.00)	0.080
TC (mmol/l)	5.32±1.00	5.14±1.00	5.42±1.00	0.081
TG (mmol/l)*	1.91 (1.25, 2.68)	1.68(1.18, 2.55)	1.98 (1.29, 2.99)	0.057
HDL-C (mmol/l)*	1.20 (1.01, 1.53)	1.34(1.03, 1.64)	1.17 (1.01, 1.50)	0.290
LDL-C (mmol/l)	2.77±0.62	2.64±0.68	2.83±0.58	0.048
UA (umol/l)	306.83±94.06	273.56±75.85	323.94±98.34	0.008
Creatinine (umol/l)	65.36±15.54	63.34±15.62	66.34±15.48	0.248

Abbreviations: BMI, body mass index; WC, waist circumference; ALT, alanine aminotransferase; AST, aspertate aminotransferase; TC, total cholesterol; TG, triglyceride; HDL-C, high-density lipoprotein cholesterol; LDL-C, low-density lipoprotein cholesterol; UA, uric acid.

* ALT, AST, TG and HDL were log_10_-transformed because of non-normal distribution.

### Plasma glucose, plasma insulin, estimates of insulin sensitivity, and pancreatic β-cell function during oral glucose tolerance test (OGTT)

The comparisons of glycemic status, insulin sensitivity, and β-cell function between the groups are summarized in Table 2. Although the levels of HbA1c were not significantly different between the two groups, 120 min blood glucose (BG_120min_) during OGTT was significantly higher in underweight or normal-weight patients than in overweight or obese patients. Compared with overweight or obese patients, underweight or normal-weight patients exhibited better insulin sensitivity and poorer pancreatic β-cell function.

**Table 2:** Insulin sensitivity, β-cell function and glucose fluctuations of the study patients

Characteristics	Total (*n*=169)	Underweight or Normal-weight (BMI<24, *n*=57)	Overweight or Obesity (BMI≥24, *n*=112)	*P*-Value
OGTT-BG 0min (mmol/l)	10.22±2.20	10.11±2.45	10.28±2.05	0.696
OGTT-BG 30min (mmol/l)	15.82±2.82	15.92±2.81	15.76±2.84	0.757
OGTT-BG 120min (mmol/l)	21.41±4.42	22.79±4.78	20.58±3.99	0.008
OGTT-Insulin 0min (μIU/ml)*	6.53 (4.06, 9.21)	5.43 (3.23, 7.45)	7.23 (4.99, 10.37)	0.004
OGTT-Insulin 30min (μIU/ml)*	13.55 (9.25, 20.25)	11.45 (8.24, 15.89)	15.16 (9.76, 22.46)	0.020
OGTT-Insulin 120min (μIU/ml)*	23.31 (14.86, 34.76)	19.21 (11.45, 26.60)	25.62 (17.30, 38.47)	0.047
HbA1c (%)	8.98±1.24	9.17±1.28	8.89±1.22	0.172
HOMA-IR*	2.76 (1.89, 4.54)	2.56 (1.59, 3.41)	3.23 (2.32, 4.75)	0.003
Matusuda ISI*	79.48 (56.52, 108.07)	92.09 (72.50, 156.31)	73.96 (49.67, 99.45)	0.003
HOMA-β*	19.96 (12.60, 31.34)	14.61 (11.16, 26.60)	24.19 (13.58, 32.93)	0.035
Insulinogenic index*	1.22 (0.51, 2.49)	1.02 (0.58, 2.01)	1.48 (0.50, 2.70)	0.444
ISSI-2*	92.67 (69.79, 110.73)	78.13 (55.07, 99.18)	100.88 (82.98, 112.34)	0.002
24h-MBG (mmol/l)	11.18±2.24	11.67±2.58	10.93±2.01	0.016
MAGE (mmol/l)	6.00±2.21	6.64±2.38	5.67±2.05	0.007
SDBG (mmol/l)	2.41±0.89	2.64±0.98	2.29±0.82	0.017
AUC_gluc>10mmol/l_ (mmol/l per day)*	1.50 (0.50, 3.10)	2.20 (0.90, 4.00)	1.35 (0.50, 2.90)	0.038
MaxBG (mmol/l)	16.85±3.39	17.59±3.59	16.47±3.23	0.043
MinBG (mmol/l)	6.80±2.05	7.00±2.32	6.69±1.90	0.359
Pre-breakfast BG (mmol/l)	9.14±2.34	9.35±2.69	9.02±2.14	0.398
PPBG peak-breakfast (mmol/l)	16.26±3.48	17.07±3.62	15.83±3.34	0.030
PPGE of breakfast (mmol/l)	7.11±2.56	7.72±2.79	6.79±2.40	0.028
Pre-lunch BG (mmol/l)	9.48±2.74	9.80±3.12	9.32±2.53	0.328
PPBG peak-lunch (mmol/l)	14.71±3.41	15.34±3.44	14.40±3.37	0.115
PPGE of lunch (mmol/l)	5.22±2.50	5.53±2.70	5.07±2.40	0.285
Pre-dinner BG (mmol/l)	9.07±2.67	9.49±2.72	8.84±2.63	0.145
PPBG peak-dinner (mmol/l)	14.33±3.43	15.45±3.71	13.72±3.12	0.002
PPGE of dinner (mmol/l)	5.26±2.46	5.96±2.24	4.87±2.50	0.008

Abbreviations: OGTT, oral glucose tolerance test; BG, blood glucose; HbA1c, glycated hemoglobin; HOMA-IR, homeostasis model assessment for insulin resistance; Matusuda ISI, Matsuda Insulin Sensitivity Index; HOMA-β, homeostasis model assessment for islet β -cell function index; ISSI-2, insulin secretion sensitivity index-2; 24h-MBG, 24-h mean blood glucose; MAGE, mean amplitude of glycemic excursions; SDBG, standard deviation of blood glucose; AUC_gluc>10mmol/L,_ area under the curve of blood glucose above 10.0 mmol/L; PPBG, postprandial blood glucose; PPGE, postprandial glucose excursion.

* OGTT-Insulin 0min, OGTT-Insulin 30min, OGTT-Insulin 120min, HOMA-IR, Matusuda ISI, HOMA-β, Insulinogenic index, ISSI-2, and AUC_gluc>10mmol/L_ were log_10_-transformed because of non-normal distribution.

### Glycemic variability and CGM measurements

**Figure 1:**
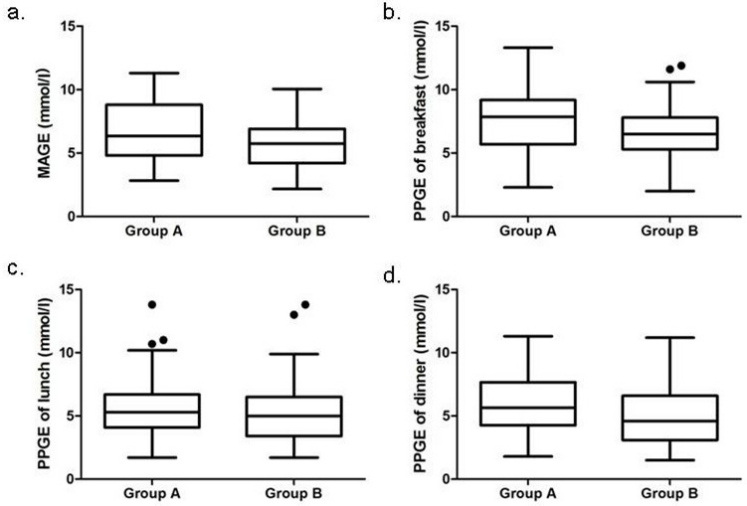
Comparison of MAGE and PPGEs between underweight or normal-weight patients (Group A) and overweight or obese patients (Group B): using box-and-whisker plot Abbreviations: MAGE, mean amplitude of glycemic excursions; PPGEs, postprandial glucose excursions. The box contained 50% of all values (from 25th to 75th percentile) and was divided by the horizontal bar of the median value (50th percentile). The whiskers showed the remainder of the distribution (1.5 × Inter Quartile Range). Outliers were shown as dots.

Table 2 and Figure 1 also show the results of CGM measurements. Compared with overweight or obese patients, underweight or normal-weight patients had significantly higher levels of the mean amplitude of glycemic excursions (MAGE) (6.64 ± 2.38 mmol/L *vs*. 5.67 ± 2.05 mmol/L; *P* = 0.007), standard deviation of blood glucose (SDBG), 24-h mean blood glucose (MBG), MaxBG, and area under the curve of blood glucose above 10.0 mmol/L (AUC_gluc > 10mmol/L_). Underweight or normal-weight patients had higher levels of postprandial glucose excursions (PPGEs) than overweight or obese patients (PPGE at breakfast, 7.72 ± 2.79 mmol/L *vs*. 6.79 ± 2.40 mmol/L, *P* = 0.028; PPGE at lunch, 5.53 ± 2.70 mmol/L *vs*. 5.07 ± 2.40 mmol/L, *P* = 0.285; PPGE at dinner, 5.96 ± 2.24 mmol/L *vs*. 4.87 ± 2.50 mmol/L, *P* = 0.008).

### Relationship between BMI and glycemic characteristics

**Figure 2:**
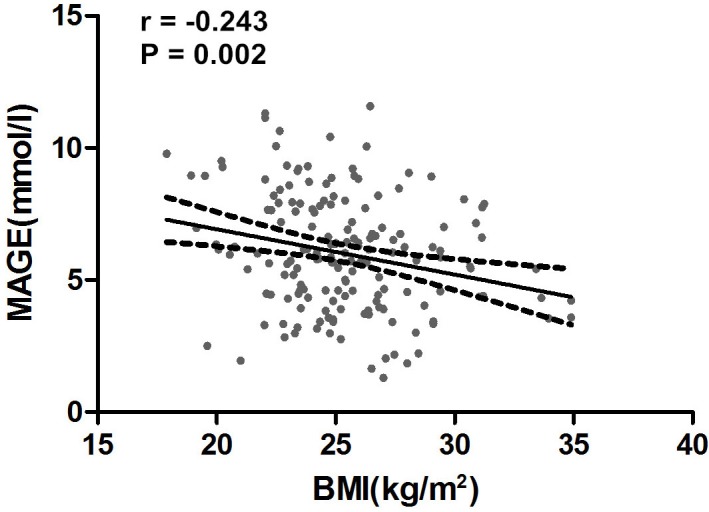
The relation between BMI and MAGE Abbreviations: BMI, body mass index; MAGE, mean amplitude of glycemic excursions.Linear relation between BMI and MAGE was observed. The correlation coefficient was −0.243 (*P* = 0.002).

Upon evaluating the correlation between BMI and glycemic characteristics (Table 3), we observed that BMI was positively correlated with homeostasis model assessment for islet beta β-cell function index (HOMA-β), insulin secretion sensitivity index-2 (ISSI-2) and homeostasis model assessment for insulin resistance (HOMA-IR); whereas negatively correlated with Matsuda Insulin Sensitivity Index (Matsuda ISI), MAGE (*r* = −0.243, *P* = 0.002, Figure 2), 24h-MBG, SDBG, AUC_gluc > 10mmol/L_, MaxBG.

**Table 3:** Linear correlation analysis of BMI and glycemic characteristics

Variables	R	*P*-value
OGTT-BG 120min	−0.228	0.013
Log_10_ OGTT- Insulin 0min*	0.370	<0.001
Log_10_ OGTT- Insulin 30min*	0.311	0.001
Log_10_ OGTT- Insulin 120min*	0.223	0.016
Log_10_ HOMA-IR*	0.368	<0.001
Log_10_ Matusuda ISI*	−0.373	<0.001
Log_10_ HOMA-β*	0.304	0.001
Log_10_ Insulinogenic index*	0.179	0.053
Log_10_ ISSI-2*	0.244	0.008
24h-MBG (mmol/l)	−0.205	0.008
MAGE (mmol/l)	−0.243	0.002
SDBG (mmol/l)	−0.190	0.014
Log_10_ AUC_gluc>10mmol/L_*	−0.240	0.002
MaxBG (mmol/l)	−0.182	0.019

Abbreviations: OGTT, oral glucose tolerance test; BG, blood glucose; HOMA-IR, homeostasis model assessment for insulin resistance; Matusuda ISI, Matsuda Insulin Sensitivity Index; HOMA-β, homeostasis model assessment for islet β -cell function index; ISSI-2, insulin secretion sensitivity index-2; 24h-MBG, 24-h mean blood glucose; MAGE, mean amplitude of glycemic excursions; SDBG, standard deviation of blood glucose; AUC_gluc>10mmol/L_, area under the curve of blood glucose above 10.0 mmol/L.

R, correlation coefficient

* OGTT-Insulin 0min, OGTT-Insulin 30min, OGTT-Insulin 120min, HOMA-IR, Matusuda ISI, HOMA-β, Insulinogenic index, ISSI-2, and AUC_gluc>10mmol/L_ were log_10_-transformed because of non-normal distribution.

### Relationship between glycemic variability and other variables

Furthermore, correlation analyses (Table 4) revealed that MAGE was negatively correlated with BMI, Matsuda ISI, HOMA-β, and ISSI-2, but was positively correlated with HbA1c, 24h-MBG, SDBG, AUC_gluc > 10mmol/L_, MaxBG, and PPGEs; however, MAGE was not associated with HOMA-IR and insulinogenic index.

**Table 4:** Linear correlation analysis of MAGE and other variables

Variables	R	*P*-value
BMI	−0.243	0.002
HbA1c	0.268	<0.001
Log10 Matusuda ISI*	−0.239	0.009
Log10 HOMA- β *	−0.218	0.018
Log10 ISSI-2*	−0.303	0.001
24h-MBG	0.414	<0.001
SDBG	0.835	<0.001
Log10 AUC_gluc>10mmol/L_ *	0.385	<0.001
MaxBG	0.675	<0.001
PPGE of breakfast	0.582	<0.001
PPGE of lunch	0.444	<0.001
PPGE of dinner	0.572	<0.001

Abbreviations: MAGE, mean amplitude of glycemic excursions; BMI, body mass index; HbA1c, glycated hemoglobin; Matusuda ISI, Matsuda Insulin Sensitivity Index; HOMA- β, homeostasis model assessment for islet β-cell function index; ISSI-2, insulin secretion sensitivity index-2; 24h-MBG, 24-h mean blood glucose; SDBG, standard deviation of blood glucose; AUC_gluc>10mmol/L,_ area under the curve of blood glucose above 10.0 mmol/L; PPGE, postprandial glucose excursion.

R, correlation coefficient

*Matusuda ISI, HOMA- β, ISSI-2 and AUC_gluc>10mmol/L_ were log_10_-transformed because of non-normal distribution.

### Multiple linear regression analysis with glycemic variability (MAGE) as the dependent variable

On multivariate regression analyses using models 1-4 (Table 5), BMI emerged as an independent variable associated with MAGE. As shown in model 3, BMI and HbA1c were two independent predictors of MAGE, but Matsuda ISI was not. Addition of the covariate ISSI-2 in model 2 revealed that BMI and ISSI-2 were two independent predictors (model 4).

**Table 5:** Multiple linear regression models of MAGE (dependent variable)

Model	Standardized β	*P*-value
Model 1:		
Age	−0.077	0.328
Sex	0.015	0.849
Family history of T2DM	0.097	0.212
BMI	−0.237	0.003
Model 2: Model 1 + HbA1C		
Age	−0.021	0.789
Sex	0.015	0.877
Family history of T2DM	0.100	0.187
BMI	−0.206	0.007
HbA1C	0.249	0.001
Model 3: Model 2 + Log10 Matusuda Index		
Age	0.048	0.611
Sex	0.013	0.890
Family history of T2DM	−0.002	0.980
BMI	−0.206	0.029
HbA1C	0.249	0.041
Log10 Matusuda Index*	0.112	0.288
Model 4: Model 2 + ISSI-2		
Age	0.070	0.750
Sex	−0.036	0.686
Family history of T2DM	−0.027	0.764
BMI	−0.231	0.013
HbA1C	0.130	0.214
Log10 ISSI-2*	−0.204	0.048

Abbreviations: MAGE, mean amplitude of glycemic excursions; BMI, body mass index; HbA1c, glycated hemoglobin; Matusuda ISI, Matsuda Insulin Sensitivity Index; ISSI-2, insulin secretion sensitivity index-2.

MAGE is regarded as the dependent variable and independent variables in model 1 included age, sex, family history of T2DM, and BMI. Furthermore, analyses were performed with the addition of the following covariates: HbA1c (model 2); HbA1c and Matsuda ISI (model 3); HbA1c and ISSI-2 (model 4).

β, regression coefficient.

*Matusuda ISI and ISSI-2 were log_10_-transformed because of non-normal distribution.

## DISCUSSION

In this study, we evaluated the glycemic variations in newly diagnosed Chinese T2DM patients with different BMI. We observed that underweight or normal-weight patients had poorer pancreatic β-cell function, and higher levels of blood glucose fluctuation parameters (MAGE, SDBG, and PPGEs) than overweight or obese patients. Moreover, our results indicated that BMI and pancreatic β-cell function were significantly associated with glycemic variability (MAGE). These findings suggested that decreased pancreatic β-cell function may contribute to increased glucose fluctuation in newly diagnosed T2DM individual with relatively low BMI. To the best of our knowledge, this was the first study to investigate the association between BMI and glycemic variability in this population.

A cross-sectional study [11] including 53 individuals with normal glucose tolerance (NGT), 53 subjects with impaired glucose regulation (IGR) and 56 T2DM patients showed that MAGE were significantly higher in IGR subjects (3.33 ± 1.55 mmol/L) and T2DM patients (4.82 ± 1.70 mmol/L) than NGT subjects (2.74 ± 1.18 mmol/L). Another cross-sectional study [12] about glycemic variability on 434 healthy Chinese adults reported that the 95th percentiles of MAGE and SDBG were 3.86 and 1.40 mmol/L, respectively. Therefore, MAGE < 3.9 mmol/L and SDBG < 1.4 mmol/L are recommended as the normal reference ranges for glycemic variability in Chinese adults. In our populations studied, MAGE and SDBG were 6.00 ± 2.21 mmol/L and 2.41 ± 0.89 mmol/L, which were much higher than the normal reference ranges for glycemic variability.

A series of previous studies [13] have revealed that HbA1c positively associate with glycemic variability in patients with T2DM. In order to exclude the influence of HbA1c on glycemic variability, we chose newly-diagnosed T2DM inpatients as study subjects. Based on our results, the levels of HbA1c in underweight or normal-weight patients were comparable to those in overweight or obese patients, but underweight or normal-weight patients had poorer pancreatic β-cell function and higher glycemic variability parameters. In agreement with our results, a cross-sectional study [6] on 521 Chinese diabetic subjects reported that BMI correlated positively with fasting C-peptide (*r* = 0.250, *P* < 0.001); underweight patients had the lowest C-peptide, whereas overweight patients had the highest C-peptide. In addition, a series of previous studies [14–16] indicated that β-cell dysfunction was related to increased glycemic variability. A cross-sectional study [14] of 59 patients with T2DM showed that postprandial β-cell function (a model-based method from plasma C-peptide and plasma glucose during a mixed meal test) was an independent contributor to glycemic variability (MAGE). Similarly, decreased oral disposition index on OGTT was associated with glycemic variability parameters across the range of glucose tolerance, from normal to pre-diabetes to T2DM [15]. Moreover, a prospective study [16] on 61 diabetic patients demonstrated that improvement of β-cell function, reflected as ISSI-2, was the main contributor for the decrease in glycemic variability. Corresponding with these studies, our results revealed that β-cell function was negatively associated with glycemic variability (MAGE). Therefore, we assumed that decline of β-cell function may be the main cause of the increased glycemic variability in newly diagnosed T2DM with relatively low BMI.

A large number of clinical studies [17–19] have indicated that postprandial hyperglycemia was independently related to cardiovascular disease, microvascular events, cognitive dysfunction, and cancer. Increased PPGEs lead to oxidative stress, inflammation, and impaired endothelial function, all of which are involved in the microvascular and macrovascular complications of diabetes [20]. Therefore, evaluation of postprandial blood glucose (PPBG) and PPGEs are particularly meaningful in patients with diabetes. In our study, we found that underweight or normal-weight patients had higher peak levels of PPBG and PPGEs than overweight or obese patients. The quantity of carbohydrate has been shown to be a consistent predictor of PPBG concentrations [21]. In addition, the type or source of carbohydrate also plays an important role in regulating PPBG levels. Wolever and Mehling [22] examined the long-term effect of varying the type of dietary carbohydrate on PPBGs in 34 subjects with impaired glucose tolerance. Their results showed that PPBGs were lowered by the same amount on high-carbohydrate, low-glycemic index diets when compared with values in subjects on the high-carbohydrate, high-glycemic index diet. However, whether there is a relationship between glycemic index or glycemic load of diets and the development of diabetes or obesity has not been determined [23–27]. To exclude the effect of diet on PPBG fluctuations, our patients ate the standardized meals with consistent compositions and sources during the CGM period. In addition, the increase of PPBG could have also been affected by impaired postprandial insulin secretion, accompanied by increased glucagon secretion and decreased hepatic and peripheral glucose uptake [28]. In our study, we found that underweight or normal-weight patients had poorer β-cell function compared with overweight or obese patients; this could be the main cause of the higher levels of PPBG and PPGEs in lower BMI patients. The levels of hormones such as glucagon and incretins, which contribute to the regulation of PPGEs, were not assessed in our study. In the future, we will evaluate whether the levels of these hormones are different between the two groups.

In our study, we found that the PPGE was higher at breakfast than at lunch or dinner in both groups. In agreement with our results, Sylvia et al. [20] have demonstrated that for the same carbohydrate intake, PPGE was 1.60 times greater at breakfast than at lunch and was 1.35 times greater at breakfast than at dinner. Another study [29] showed that the AUC 1-4 h after a meal was significantly larger after breakfast compared with the values after lunch and dinner. The higher PPGE after breakfast may be due to the “dawn phenomenon”, which resulted in increased hepatic glucose output and decreased glucose utilization in the morning.

Some limitations of this study deserve comments. First, we estimated insulin secretion and insulin sensitivity based on the OGTT, not by the “gold standard” test, which is the glucose-clamp technique; however, clamping is expensive and not easily feasible in a relatively large-scale study, and we believe that proxy measures are reliable. A previous study [30] has indicated that Matsuda ISI was positively related to clamp value (*r* = 0.77, *P* < 0.001). ISSI-2 has been directly validated against the disposition index from the intravenous glucose tolerance test and has been used to measure β-cell function in several previous studies [16, 31]. Second, we combined overweight and obese patients in one study group because of the relatively small sample size of obese patients. Third, hormones (i.e., incretin and glucagon) that may contribute to the regulation of PPGEs and be relevant to glycemic variability were not assessed in our study.

Despite these potential limitations, this study had several advantages. First, glycemic variability was assessed by CGM in a relatively large population. Second, to minimize confounding factors, we selected newly diagnosed and drug-naïve T2DM as study subjects who were controlled for consistent physical activity and diets during the CGM period.

In this study on Chinese patients with newly diagnosed T2DM, patients with relatively low BMI had increased glycemic variability characterized by elevated PPGEs. Moreover, poor β-cell function might be the main contributory factor associated with increased glycemic variability in patients with lower BMI. Our data suggested that improvement of β-cell function and reduction of postprandial hyperglycemia could be important therapeutic targets in controlling glycemic variability in T2DM patients with lower BMI. A prospective study is warranted to validate whether underweight or normal-weight patients with T2DM are more likely to develop diabetes complications than overweight or obese patients with T2DM in China.

## MATERIALS AND METHODS

### Study patients

Newly diagnosed T2DM patients who visited the Department of Endocrinology of Nanjing First Hospital between June 2014 and November 2015 were enrolled in this study. Inclusion criteria were age > 18 years and newly diagnosed and drug-naïve T2DM. Exclusion criteria were as follows: 1) presence of liver or renal dysfunction (liver enzyme levels > 2.5-fold the upper normal limit or serum creatinine > 1.3-fold the upper normal limit, respectively); 2) use of medications or drugs that may alter glucose metabolism (e.g., thyroid hormones, glucocorticoids, and thiazide diuretics); 3) presence of islet autoantibodies, such as glutamic acid decarboxylase autoantibody, islet cell antibody, insulin autoantibody or insulinoma-associated protein 2 autoantibody; 4) presence of acute or chronic infectious disease, pregnancy or lactation, or a history of cancer. Finally, 169 patients (110 men and 59 women, mean age of 51.33 ± 9.83 years) were analyzed in this study. This study was approved by the ethics committee of Nanjing First Hospital and all study patients gave written informed consent. The methods were carried out in accordance with the Declaration of Helsinki guidelines, including any relevant details.

### Clinical and laboratory assessments

The social-behavioral information, family and medical histories, and health-related habits were checked by the study physicians. Smoking or alcohol consumption was defined as daily cigarette or alcohol use, respectively, for at least 12 months, regardless of the amount. Anthropometric measurements, including height, weight, and WC; systolic blood pressure (SBP); and diastolic blood pressure (DBP) were determined by experienced nurses. WC was measured at the midpoint between the lower border of the rib cage and the iliac crest. BMI was calculated as weight divided by the square of height (kg/m2). According to the Working Group on Obesity in China in 2003 [32], we classified patients as underweight or normal (BMI < 24 kg/m2) and overweight or obese (BMI ≥ 24 kg/m2). Peripheral blood pressure was measured using brachial sphygmomanometry and was recorded as the mean of three measured readings. Patients with a history of hypertension or abnormally high arterial blood pressure (SBP ≥ 140 mmHg or DBP ≥ 90 mmHg) were considered as hypertension.

After at least 10 h of overnight fast, all study patients were subjected to a 75-g OGTT and blood samples were collected at 0, 30, and 120 min. Plasma blood glucose levels were measured using hexokinase method. Serum insulin concentrations were measured using radioimmunoassay (Beijing Technology Company; Beijing, China). Insulin sensitivity was evaluated by HOMA-IR [HOMA-IR = fasting blood insulin (FBI) (mU/L) × fasting blood glucose (FBG) (mmol/L) / 22.5] [33] and Matsuda ISI [Matsuda ISI = 10,000 / (FBG × FBI × mean glucose during OGTT × mean insulin during OGTT)1/2] [34]. β-cell function was estimated by HOMA-β [HOMA-β = 20 × FBI / (FBG - 3.5)] [33]; insulinogenic index [Δinsulin30 / Δglucose30 = (insulin30 - FBI) / (glucose30 - FBG)] [35] and ISSI-2 [36]. Area under the insulin curve (AUC_ins_) and area under the glucose curve (AUC_gluc_) were calculated using the trapezoidal rule [37], ISSI-2 = (AUC_ins_/AUC_gluc_) × Matsuda ISI. Plasma total cholesterol (TC), triglyceride (TG), high-density lipoprotein cholesterol (HDL-C), LDL-C, ALT, aspartate aminotransferase (AST), blood urea nitrogen (BUN), creatinine and UA were measured with an auto-analyzer (Modular E170; Roche, Mannheim, Germany). HbA1c levels were determined using high-performance liquid chromatography (BIO-RAD Company; Hercules, CA, USA).

After collection of clinical and laboratory data, a CGM device was placed in all study patients for 3 days. The CGM sensor was inserted on day 0 at 4:00pm - 5:00pm. and was removed on day 4 at 8:00am - 9:00am. Every day during the CGM period, capillary blood glucose was checked at least four times to calibrate the glucose values of the CGM sensor. During the period of CGM sensor monitoring, all patients were required to refrain from both structured and recreational physical activity. Patients were provided with a standardized diet, which was designed to ensure a total daily energy intake of 105 KJ/kg/day consisting of 55% of energy from carbohydrate, 18% from protein, and 27% from fat. The foods were divided into three equal portions consumed by patients at 07:00am - 8:00am, 11:30am - 12:30am and 5:30pm - 6:30pm. The CGM indices adopted in this study included MAGE, 24-h MBG, SDBG, AUC_gluc > 10mmol/L_, and PPGEs. PPGE was calculated as the peak glucose value after meals minus the glucose level at the beginning of each meal [11].

### Statistical analysis

All statistical analyses were conducted using SPSS 17.0 for Windows (Chicago, IL, USA). Continuous variables were tested for normality of distribution by one-sample Kolmogorov-Smirnov tests, and natural log transformations of abnormally distributed variables were used as necessary. Variables with normal distribution were expressed as mean ± standard deviation, those with abnormal distribution were expressed as median (interquartile range), and categorical variables were expressed as proportion. After assessing the homogeneity of variances, we evaluated the differences in clinical, biochemical values, insulin sensitivity, β-cell function, and glucose fluctuations between underweight or normal-weight patients and overweight or obese patients using Student's *t*-test, Welch's *t*-test or chi-square test, as appropriate. The associations between BMI, glucose variability (MAGE) and other various parameters were assessed by Pearson correlation analyses. Multiple linear regression analyses of MAGE (dependent variable) were performed with model 1, which consisted of the following variables: age, sex, family history of T2DM and BMI. Furthermore, analyses were performed with the addition of the following covariates: HbA1c (model 2); HbA1c and Matsuda ISI (model 3); HbA1c and ISSI-2 (model 4). The statistical tests were two-sided and a P-value of < 0.05 was considered statistically significant.
